# Oestrogenic activity of tamoxifen and its metabolites on gene regulation and cell proliferation in MCF-7 breast cancer cells.

**DOI:** 10.1038/bjc.1989.153

**Published:** 1989-05

**Authors:** M. D. Johnson, B. R. Westley, F. E. May

**Affiliations:** University Department of Pathology, Royal Victoria Infirmary, Newcastle upon Tyne, UK.

## Abstract

The effects of tamoxifen, three of its in vivo metabolites and 3-hydroxytamoxifen on cellular proliferation and the induction of four oestrogen-regulated RNAs (pNR-1, pNR-2, pNR-25 and cathepsin D) have been measured in MCF-7 breast cancer cells in phenol red-free culture medium. Tamoxifen and 3-hydroxytamoxifen acted as partial oestrogens to stimulate cell growth and the levels of the pNR-2 and pNR-25 RNAs. They were full oestrogens for the induction of cathepsin D RNA and induced the pNR-1 RNA above the level found in oestrogen-treated cells. N-Desmethyltamoxifen and 4-hydroxytamoxifen behaved like tamoxifen except that N-desmethyltamoxifen did not induce the pNR-2 RNA and was only a partial oestrogen for the induction of cathepsin D RNA, and 4-hydroxytamoxifen did not induce the pNR-2 or pNR-25 RNAs. In the presence of oestradiol, the four anti-oestrogens prevented the stimulation of growth and reduced (pNR-2 and pNR-25) or increased (pNR-1) the RNA levels to those present in MCF-7 cells treated with the anti-oestrogen alone. In contrast, for cathepsin D RNA levels there was a synergistic effect of the anti-oestrogens and oestradiol. The concentration at which each anti-oestrogen was effective was related to its affinity for the oestrogen receptor. Metabolite E was a full oestrogen for the induction of cell proliferation and the oestrogen-regulated RNAs. pNR-25 and pNR-2 RNA levels correlated most closely with effects on cell proliferation. These RNAs are therefore potentially the most useful for predicting the response of breast cancer patients to tamoxifen therapy.


*Br. J. Cancer* (1989), **59**, 727–738 © The Macmillan Press Ltd., 1989

### Oestrogenic activity of tamoxifen and its metabolites on gene regulation and cell proliferation in MCF-7 breast cancer cells

M.D. Johnson, B.R. Westley & F.E.B. May

*University Department of Pathology, Royal Victoria Infirmary, Newcastle upon Tyne NE1 4LP, UK.*

**Summary** The effects of tamoxifen, three of its *in vivo* metabolites and 3-hydroxytamoxifen on cellular proliferation and the induction of four oestrogen-regulated RNAs (pNR-1, pNR-2, pNR-25 and cathepsin D) have been measured in MCF-7 breast cancer cells in phenol red-free culture medium. Tamoxifen and 3-hydroxytamoxifen acted as partial oestrogens to stimulate cell growth and the levels of the pNR-2 and pNR-25 RNAs. They were full oestrogens for the induction of cathepsin D RNA and induced the pNR-1 RNA above the level found in oestrogen-treated cells. *N*-Desmethyltamoxifen and 4-hydroxytamoxifen behaved like tamoxifen except that *N*-desmethyltamoxifen did not induce the pNR-2 RNA and was only a partial oestrogen for the induction of cathepsin D RNA, and 4-hydroxytamoxifen did not induce the pNR-2 or pNR-25 RNAs. In the presence of oestradiol, the four anti-oestrogens prevented the stimulation of growth and reduced (pNR-2 and pNR-25) or increased (pNR-1) the RNA levels to those present in MCF-7 cells treated with the anti-oestrogen alone. In contrast, for cathepsin D RNA levels there was a synergistic effect of the anti-oestrogens and oestradiol. The concentration at which each anti-oestrogen was effective was related to its affinity for the oestrogen receptor. Metabolite E was a full oestrogen for the induction of cell proliferation and the oestrogen-regulated RNAs. pNR-25 and pNR-2 RNA levels correlated most closely with effects on cell proliferation. These RNAs are therefore potentially the most useful for predicting the response of breast cancer patients to tamoxifen therapy.

Breast cancer is a common disease that will often respond to endocrine therapy. Tamoxifen is the most frequently used endocrine therapy for post-menopausal women (Furr & Jordan, 1984). Remission is obtained in a third of all patients and in 50% of patients whose tumours contain oestrogen receptors. However, many patients do not respond and among those that do, the average time to relapse is only 14 months. Although tamoxifen is thought to act through the oestrogen receptor, its mechanism of action is not fully understood (Furr & Jordan, 1984).

Tamoxifen is extensively metabolised *in vivo* and it is therefore important to understand the action of the metabolites as well as that of the parent drug. In breast cancer patients, *N*-desmethyltamoxifen and 4-hydroxytamoxifen are the major metabolites found in plasma and tumour cell extracts (Robinson & Jordan, 1988). Among other minor metabolites found in patient plasma, metabolite E is of note as it has been reported to be oestrogenic in rat uterus and pituitary (Murphy *et al.*, 1987; Robinson & Jordan, 1988).

Several cell lines have been isolated from metastatic breast cancer cells that express oestrogen receptor and whose growth is stimulated by oestrogens (e.g. MCF-7: Soule *et al.*, 1973; Horwitz *et al.*, 1978; Lippman *et al.*, 1976). In previous studies on the effects of tamoxifen and its metabolites on growth (e.g. Coezy *et al.*, 1982) cells were cultured in medium containing the pH indicator dye phenol red, which is a weak oestrogen (Berthois *et al.*, 1986). Under these conditions tamoxifen only inhibited cell growth. A reappraisal of the effects of tamoxifen and its metabolites in phenol red-free medium is therefore necessary, especially as tamoxifen has been reported to stimulate the proliferation of MCF-7 cells grown in phenol red-free medium (Berthois *et al.*, 1986).

A number of oestrogen-regulated RNAs have been isolated recently from MCF-7 and ZR 75 cells (May & Westley, 1986, 1988; Westley & May, 1987). The pNR-2 RNA corresponds to the pS2 RNA identified by others (Masiakowski *et al.*, 1982). It codes for a small cysteine rich protein which is secreted by normal stomach mucosa (Rio *et al.*, 1988) and has structural similarities to IGF-1. It is present in some breast tumours and appears to be regulated by oestrogen in the tumour cells (Rio *et al.*, 1987). The pNR-100 RNA codes for the lysosomal aspartyl protease cathepsin D (Westley & May, 1987). The proteins encoded by the pNR-1 and pNR-25 RNAs have not been identified but the pNR-1 RNA is of interest because it is induced by tamoxifen and 4-hydroxytamoxifen (May & Westley, 1987).

In this study, the oestrogenic and antioestrogenic activities of tamoxifen, *N*-desmethyltamoxifen, 4-hydroxytamoxifen, 3-hydroxytamoxifen and metabolite E have been measured on cell proliferation and on the induction of the oestrogen-regulated pNR-1, pNR-2, pNR-25 and pNR-100 RNAs in phenol red-free medium. The antioestrogens had widely differing effects on cell proliferation; 4-hydroxytamoxifen was the least oestrogenic while metabolite E was fully oestrogenic.

### Materials and methods

### *Cell culture*

MCF-7 cells were maintained in Dulbecco's modified Eagle's medium containing 10% foetal calf serum and insulin ($1\,\mu g\,ml^{-1}$). Charcoal-treated newborn calf serum was used in all experiments designed to test the effects of oestrogens and anti-oestrogens on cell growth or on the levels of the oestrogen regulated RNAs. Newborn calf serum was used because it contains lower concentrations of steroids than fetal calf serum. This enables cells to be withdrawn more effectively from the steroids present in normal growth medium and facilitates the analysis of small agonist effects of antioestrogens.

For analysis of RNA regulation, cells were grown in T25 flasks. They were then withdrawn from the steroids present in the routine culture medium by culture for 7 days in phenol red-free modified Eagle's medium containing 10% newborn calf serum, treated with dextran-coated charcoal to remove endogenous steroids, and insulin ($1\,\mu g\,ml^{-1}$). During the first 3 days of culture the cells were washed twice before the medium change. Withdrawn cells were then cultured continuously in the above medium, whereas treated cells were cultured in the above medium supplemented with the indicated hormone. During withdrawal and treatment, culture medium was changed daily. For measurement of cell growth, cells were plated into 16-mm wells at approximately 10% confluence and allowed to attach overnight. They were withdrawn from endogenous steroids essentially as described

Correspondence: F.E.B. May.
Received 10 November 1988, and in revised form, 4 January 1989.

above except that for the first 3 days the medium was changed twice daily and the cells were not washed with PBS. On the fifth day, the indicated drug or solvent alone were added to the culture medium and thereafter medium was changed daily.

For the measurement of cathepsin D synthesis, cells were plated into 8-mm wells, withdrawn from the endogenous steroids present in the maintenance medium and treated with various concentrations of oestradiol and tamoxifen as described for the analysis of the regulated RNAs. Cells were labelled with $^{35}$S-methionine (200 $\mu$Ci ml$^{-1}$) for 1 or 6 h as described by Westley & Rochefort (1980) and cell extracts prepared for immunoprecipitation according to Buetti & Diggelmann (1981).

*Preparation and analysis of RNA*

Total RNA was prepared, denatured, electrophoresed through denaturing agarose gels and transferred to nitrocellulose or nylon membranes as described by May & Westley (1987). $^{32}$P-labelled probes were synthesised by transcription from cDNA subcloned into pGEM and Bluescript vectors. Hybridisation to the immobilised RNA was at 65°C in a solution containing 50% formamide (Melton *et al.*, 1984). The amount of radiolabelled probe hybridised was quantified by densitometric scanning of the autoradiographs.

*DNA measurement*

The cells were lysed in a solution of triton X-100 and ammonia and the DNA measured using bisbenzimidazole (Hoechst 33258) on a Kontron SFM 25 spectrofluorometer (Downs & Wilfinger, 1983). The DNA content of one of the wells treated with oestradiol for 9 days was taken as the 100% value for each experiment. Triplicate wells were analysed for each time point.

*Immunoprecipitation*

$^{35}$S-labelled cellular proteins (200,000 c.p.m.) were immunoprecipitated with mono-specific polyclonal rabbit antisera against human cathepsin D (Reid *et al.*, 1986) using protein-A sepharose. Immunoprecipitates were analysed on 12% polyacrylamide gels which were processed for fluorography and exposed to prefogged X-ray film at −70°C. The incorporation of $^{35}$S-methionine into cathepsin D was measured by densitometric scanning of the X-ray films (Laskey & Mills, 1975).

### Results

*Oestrogen-dependent growth of MCF-7 cells*

To optimise the conditions for maximal oestrogenic stimulation of MCF-7 cell growth, several MCF-7 sublines were tested. Although the growth of all sublines was stimulated, the subline chosen for this study showed the greatest stimulation (usually more than 10-fold over a 9-day period) and did not detach even during periods of prolonged withdrawal in phenol red-free medium.

The effect of oestrogen on the proliferation of these MCF-7 cells is shown in Figure 1. There was complete cessation of the growth of cells cultured continuously in the withdrawal medium alone. The stimulation of the growth by oestradiol was dose dependent. A small increase was detected in the presence of $10^{-12}$ M oestradiol and growth was maximal after the addition of $10^{-10}$ M oestradiol to the culture medium. In this experiment, there was an increase of approximately 60-fold in the DNA recovered from cells grown for 9 days in the presence of oestrogen. The dose–response curve of the growth stimulation by oestradiol after 9 days of culture in its presence indicates that the increase is half-maximal in $5 \times 10^{-12}$ M oestradiol.

*Regulation of cell growth and oestrogen-regulated RNAs by tamoxifen*

The ability of tamoxifen to influence the growth of MCF-7 cells in both the absence and presence of oestradiol was tested as described in **Materials and methods**. As in the experiment described above, there was complete cessation of the growth of cells cultured in the withdrawal medium alone whereas the addition of $10^{-8}$ M oestradiol to the culture medium promoted cell proliferation (Figure 2a). Tamoxifen at $10^{-8}$ M had no effect on the proliferation of the MCF-7 cells. However, $10^{-6}$ and $10^{-7}$ M tamoxifen stimulated cell growth.

**Figure 1** Stimulation of MCF-7 cell proliferation by oestradiol. **a**, Cells were withdrawn and then treated with various concentrations of oestradiol for the indicated lengths of time. The amount of DNA per well is expressed as a fraction of the DNA in a well of cells treated with $10^{-7}$ M oestradiol. The mean (●) and the standard error from the mean (bars) of at least three determinations are shown. **b**, The amount of DNA in cells grown for nine days in the presence of the indicated concentration of oestradiol is expressed as a percentage of the maximum level.

**Figure 2** Effect of tamoxifen on MCF-7 cells. **a**, Cells were withdrawn for 5 days and then cultured in the withdrawal medium alone (○—○), or containing $10^{-8}$ M oestradiol (∅—∅), or $10^{-8}$, $10^{-7}$ or $10^{-6}$ M tamoxifen (●—●) for the indicated lengths of time. **b**, Cells were withdrawn for 5 days and then cultured in the withdrawal medium alone (○—○) or containing $2 \times 10^{-10}$ M oestradiol alone (∅—∅) or plus $10^{-7}$, $10^{-6}$ or $10^{-5}$ M tamoxifen for the indicated length of time (●—●). The concentration of DNA in each well is expressed as a fraction of the amount in one of the oestradiol treated wells for each experiment. The values shown are the mean of three determinations plus or minus the standard error of the mean. **c**, MCF-7 cells were withdrawn for 7 days and then cultured for 3 days in the indicated concentration of tamoxifen alone (○—○) or in the presence (●—●) of $2 \times 10^{-10}$ M oestradiol. Total RNA was prepared, separated by gel electrophoresis, and transferred to a membrane support. The filters were hybridised with radiolabelled RNA probes and the amount hybridised was measured by scanning autoradiographs obtained using preflashed X-ray film exposed at −70°C. The amount of cDNA hybridised is plotted as a percentage of the amount in oestrogen-treated cells for each plasmid. The dashed lines indicate the levels of the RNAs in cells grown continuously in the withdrawal medium.

Cells grown simultaneously in the presence of $2\times10^{-10}$ M oestradiol and $10^{-7}$ M tamoxifen grew at the same rate as cells grown in the presence of $2\times10^{-10}$ M oestradiol alone (Figure 2b). Tamoxifen at $10^{-6}$ M inhibited the oestrogen-induced cell proliferation by approximately 20%. The effect of $2\times10^{-10}$ M oestradiol on MCF-7 cell growth was almost completely inhibited by $10^{-5}$ M tamoxifen.

The effect of tamoxifen on the levels of four oestrogen-induced RNAs, pNR-1, pNR-2, pNR-25 and cathepsin D, was tested in the MCF-7 cell subline used in the growth experiments (Figure 2c). In these MCF-7 cells, tamoxifen increased the level of the pNR-1 RNA to 200% of the level induced by oestradiol. The induction was dose-dependent and was half-maximal at approximately $2\times10^{-8}$ M tamoxifen. In the presence of oestradiol, tamoxifen also induced pNR-1 RNA levels above the maximal level induced by oestradiol to the level obtained in cells treated with tamoxifen alone. The additional increase was half-maximal in the presence of $4\times10^{-7}$ M tamoxifen.

Tamoxifen was much less oestrogenic for the induction of the pNR-2 RNA. Tamoxifen alone induced the pNR-2 RNA to about 30% of the level present in oestradiol treated cells. It inhibited the induction of the pNR-2 RNA by $2\times10^{-10}$ M oestradiol, to the levels found in cells treated with tamoxifen alone. The inhibition was half-maximal in the presence of $5\times10^{-6}$ M tamoxifen.

The pNR-25 RNA was induced by $10^{-6}$ M tamoxifen, to approximately 15% of the level in oestrogen-treated cells. The induction was half-maximal in the presence of $5\times10^{-8}$ M tamoxifen. The induction by oestradiol of pNR-25 RNA levels was inhibited by tamoxifen; half-maximal inhibition was achieved by $4\times10^{-6}$ M tamoxifen.

The induction of cathepsin D RNA by tamoxifen was half-maximal at $5\times10^{-8}$ M. In the presence of $10^{-6}$ M tamoxifen, cathepsin D RNA levels were the same as in oestrogen-treated cells. In the presence of $2\times10^{-10}$ M oestradiol, the effect of increasing concentrations of tamoxifen on cathepsin D RNA levels was dramatic (Figures 2c and 3). Maximal cathepsin D RNA levels of eight times the level in cells treated with oestradiol alone were induced by simultaneous treatment with $2\times10^{-10}$ M oestradiol and between $10^{-6}$ and $5\times10^{-6}$ M tamoxifen. Above $5\times10^{-6}$ M tamoxifen, cathepsin D RNA levels were lower but remained above the levels in cells treated with $2\times10^{-10}$ M oestradiol alone.

*Regulation of cell growth and oestrogen-induced RNAs by* N-*desmethyltamoxifen*

The effects of *N*-desmethyltamoxifen on the proliferation of MCF-7 cells are shown in Figure 4a. While it had no effect at $10^{-5}$ M, *N*-desmethyltamoxifen stimulated the proliferation at both $10^{-6}$ and $10^{-7}$ M. The increase in cell numbers induced by $10^{-7}$ M *N*-desmethyltamoxifen was 24% of the increase induced by oestradiol. Neither $10^{-7}$ nor $10^{-6}$ M *N*-desmethyltamoxifen had any effect on the cell proliferation induced by $2\times10^{-10}$ M oestradiol whereas $10^{-5}$ M *N*-desmethyltamoxifen almost completely inhibited the effect of oestradiol (Figure 4b).

The levels of the four oestrogen-regulated RNAs in cells grown in the presence of different concentrations of *N*-desmethyltamoxifen are shown in Figure 4c. The induction of pNR-1 RNA levels by *N*-desmethyltamoxifen both alone and in the presence of oestradiol was similar to the induction

**Figure 3** Induction of cathepsin D RNA levels by tamoxifen, *N*-desmethyltamoxifen, 4-hydroxytamoxifen and 3-hydroxytamoxifen in the presence of oestradiol. Withdrawn MCF-7 cells were cultured for 3 days in withdrawal medium containing $2\times10^{-10}$ M oestradiol together with the indicated concentration of tamoxifen (●—●), *N*-desmethyltamoxifen (○—○), 4-hydroxytamoxifen (■—■) or 3-hydroxytamoxifen (□—□). Cathepsin D RNA levels were measured as described in **Materials and methods** and are expressed as a percentage of the levels in oestrogen treated cells.

**Figure 4** Effect of *N*-desmethyltamoxifen in MCF-7 cells. **a** and **b**, Withdrawn cells were cultured in the withdrawal medium alone (○—○) or containing $10^{-8}$ M (**a**) or $2 \times 10^{-10}$ M (**b**) oestradiol (Ø—Ø), or different concentrations of *N*-desmethyltamoxifen (●—●) in the absence (**a**) or presence (**b**) of $2 \times 10^{-10}$ M oestradiol for the indicated lengths of time. The data are expressed as described in the legend to Figure 2. **c**, MCF-7 cells were withdrawn and then cultured in the indicated concentrations of *N*-desmethyltamoxifen in the presence (●—●) or absence of $2 \times 10^{-10}$ M oestradiol (○—○). The levels of the pNR-1, pNR-2, pNR-25 and cathepsin D RNAs were measured as described in the legend to Figure 2c.

by tamoxifen. Induction by *N*-desmethyltamoxifen alone was half-maximal at about $2\times10^{-8}$ M and the additional induction in the presence of oestradiol was half-maximal at about $4\times10^{-6}$ M *N*-desmethyltamoxifen.

On its own, *N*-desmethyltamoxifen had little effect on pNR-2 RNA levels, but inhibited the oestrogen-induction with half-maximal inhibition at around $10^{-5}$ M. *N*-Desmethyltamoxifen did, however, induce the pNR-25 RNA levels to approximately the same extent as tamoxifen. Maximal induction was attained in the presence of $10^{-7}$ M. *N*-Desmethyltamoxifen completely inhibited the induction of the pNR-25 RNA by oestradiol at $10^{-5}$ M. The inhibition was half-maximal at approximately $2\times10^{-6}$ M *N*-desmethyltamoxifen.

*N*-Desmethyltamoxifen was less oestrogenic than tamoxifen for the induction of cathepsin D RNA. At $10^{-7}$ M it increased cathepsin D RNA levels to approximately 40% of those in oestrogen-treated cells. In the presence of $2\times10^{-10}$ M oestradiol, there was an additive effect similar to but less dramatic than that observed with tamoxifen (Figures 3 and 4c). Maximum cathepsin D levels of 3.3 times the level in oestradiol treated cells were induced by $3\times10^{-6}$ M *N*-desmethyltamoxifen and $2\times10^{-10}$ M oestradiol.

*Regulation of cell growth and oestrogen-induced RNAs by 4-hydroxytamoxifen*

4-Hydroxytamoxifen stimulated cell proliferation at $10^{-9}$ M but had no effect at the other concentrations tested, apart from $10^{-5}$ M, which was cytotoxic (Figure 5a). At $10^{-5}$ and $10^{-6}$ M it completely inhibited the stimulation of cell proliferation induced by oestradiol. In the presence of $10^{-7}$ M 4-hydroxytamoxifen and $2\times10^{-10}$ M oestradiol, cells grew at the same rate as cells cultured in $2\times10^{-10}$ M oestradiol alone.

The induction of the pNR-1 RNA by 4-hydroxytamoxifen was, however, similar to that obtained with tamoxifen and *N*-desmethyltamoxifen. The induction by 4-hydroxytamoxifen alone was half-maximal at about $3\times10^{-10}$ M and the stimulation above the level induced by oestradiol in the presence of both drugs was half-maximal at around $4\times10^{-7}$ M 4-hydroxytamoxifen. 4-Hydroxytamoxifen alone had no effect on the levels of the pNR-2 and pNR-25 RNAs. At a concentration of $3\times10^{-7}$ M, 4-hydroxytamoxifen completely inhibited the induction by oestradiol of these two RNAs. The induction of the cathepsin D RNA by 4-hydroxytamoxifen was half-maximal at $\sim4\times10^{-10}$ M and attained the levels present in cells maximally stimulated by oestradiol. The dose–response curve for the induction of cathepsin D RNA levels by 4-hydroxytamoxifen in the presence of oestradiol was bell-shaped with a maximum at $2\times10^{-7}$ M (Figures 3 and 5c). At this concentration, the cathepsin D RNA concentration was approximately 5 times that in oestradiol-treated cells.

*Regulation of cell growth and oestrogen-induced RNAs by 3-hydroxytamoxifen*

3-Hydroxytamoxifen stimulated the proliferation of MCF-7 cells at $10^{-8}$ M but not at $10^{-5}$ or $10^{-11}$ M (Figure 6a). After 9 days' treatment, the increase induced by 3-hydroxytamoxifen was 14% of the induction by oestradiol. The cell proliferation induced by $2\times10^{-10}$ M oestradiol was not affected by $10^{-9}$ M 3-hydroxytamoxifen, but was substantially reduced by $10^{-6}$ M and was completely inhibited by $10^{-5}$ M 3-hydroxytamoxifen (Figure 6b).

The effects of 3-hydroxytamoxifen on the levels of the oestrogen-induced RNAs are shown in Figure 6c. The pNR-1 RNA was induced to 150% of the level induced by oestradiol; with half-maximal induction in the presence of $\sim1.5\times10^{-9}$ M 3-hydroxytamoxifen. In the presence of oestradiol, 3-hydroxytamoxifen induced the pNR-1 RNA levels above the level induced by oestradiol to the level obtained with 3-hydroxytamoxifen alone.

The pNR-2 and pNR-25 RNA levels were both induced by $10^{-8}$ M 3-hydroxytamoxifen to 10% and 20%, respectively, of the levels induced by oestradiol. The induction of both RNAs by oestradiol was completely inhibited by this antioestrogen. Cathepsin D RNA was induced to the levels found in oestradiol-treated cells by $10^{-6}$ M 3-hydroxytamoxifen. The induction was half-maximal in the presence of $5\times10^{-8}$ M 3-hydroxytamoxifen. The levels of cathepsin D RNA in cells treated simultaneously with $2\times10^{-10}$ M oestradiol and different concentrations of 3-hydroxytamoxifen gave a bell-shaped curve with a maximum at $3\times10^{-7}$ M 3-hydroxytamoxifen (Figures 3 and 6c).

*Regulation of cell growth and oestrogen-induced RNAs by metabolite E*

The effects of metabolite E on cell growth are shown in Figure 7a. In the presence of $10^{-9}$ M metabolite E, MCF-7 cells grew at 70% of the rate induced by $10^{-8}$ M oestradiol. Metabolite E at $10^{-7}$ M was as effective as oestradiol at stimulating the growth of these MCF-7 cells. Cells cultured in $2\times10^{-10}$ M oestradiol alone or in the presence of $10^{-5}$, $10^{-6}$ or $10^{-7}$ M metabolite E grew at the same rate.

Metabolite E was also as effective as oestradiol for the induction of the four oestrogen-regulated RNAs. Half-maximal induction was attained at $4\times10^{-10}$ M for pNR-1, $8\times10^{-9}$ M for the pNR-2, $1.5\times10^{-9}$ M for pNR-25, and $1.5\times10^{-9}$ M for cathepsin D. The RNA levels induced by metabolite E were the same as those found in oestradiol-treated cells.

*Effects of oestrogen and tamoxifen on cathepsin D synthesis*

To determine whether the increased cathepsin D RNA levels that are induced by combined oestradiol and tamoxifen treatment result in increased synthesis of cathepsin D, newly synthesised proteins were labelled with $^{35}$S-methionine for 1 h. Incorporation into cathepsin D was then measured using immunoprecipitation as described in the **Materials and methods.** A single band of approximately 46 kDa was seen, which corresponds to pro-cathepsin D. Overall, the effects of oestradiol and tamoxifen on pro-cathepsin D synthesis were very similar to the effects on its RNA (Figure 8). Labelling of pro-cathepsin D was increased 10-fold by oestradiol ($2\times10^{-10}$ M). Tamoxifen alone increased pro-cathepsin D synthesis at concentrations above $10^{-8}$ M. Maximal levels were obtained at $3\times10^{-7}$ M and these were similar to the oestradiol stimulated levels.

The synthesis of pro-cathepsin D was at the oestrogen stimulated level in the presence of oestradiol and tamoxifen together up to a concentration of $3\times10^{-7}$ M tamoxifen. At higher concentrations of tamoxifen ($10^{-6}$ to $5\times10^{-6}$ M) synthesis of pro-cathepsin D was 3.2-fold higher than in oestrogen stimulated cells. This dropped to 1.6-fold in the presence of $10^{-5}$ M tamoxifen and $2\times10^{-10}$ M oestradiol.

The secretion of cathepsin D from MCF-7 cells (treated with the same concentrations of oestradiol and tamoxifen as in the experiments described above) was measured following a 6-h labelling with $^{35}$S-methionine. The amount of cathepsin D secreted into the medium closely mirrored its synthesis (data not shown).

### Discussion

The growth of the MCF-7 cells described in this report was extremely sensitive to oestrogen. The growth stimulation was dose dependent with near maximal proliferation in the presence of $10^{-10}$ M oestradiol. The effects of tamoxifen and its derivatives varied significantly, although all five stimulated cell proliferation. 4-Hydroxytamoxifen alone stimulated cell proliferation only slightly at a single concentration ($10^{-9}$ M) and was cytotoxic at $10^{-5}$ M. Tamoxifen, *N*-desmethyltamoxifen and 3-hydroxytamoxifen all stimulated cell proliferation to a greater extent. The stimulation was dose-dependent, following a bell-shaped curve, with no effect in the presence of $10^{-5}$ M. No cytotoxic effects of high concentrations of these three anti-oestrogens were observed

**Figure 5** Effect of 4-hydroxytamoxifen in MCF-7 cells. **a** and **b**, Withdrawn cells were cultured in the withdrawal medium alone (○—○), or containing $10^{-8}$ M (**a**) or $2 \times 10^{-10}$ M (**b**) oestradiol (∅—∅), or different concentrations of 4-hydroxytamoxifen (●—●) in the absence (**a**) or presence (**b**) of $2 \times 10^{-10}$ M oestradiol for the indicated lengths of time. The data are expressed as described in the legend to Figure 2. **c**, MCF-7 cells were withdrawn and then cultured in the indicated concentrations of 4-hydroxytamoxifen in the presence (●—●) or absence of $2 \times 10^{-10}$ M oestradiol (○—○). The levels of the pNR-1, pNR-2, pNR-25 and cathepsin D RNAs were measured as described in the legend to Figure 2c.

**Figure 6** Effect of 3-hydroxytamoxifen in MCF-7 cells. **a** and **b**, Withdrawn cells were cultured in the withdrawal medium alone (O—O), or containing $10^{-8}$ M (**a**) or $2 \times 10^{-10}$ M (**b**) oestradiol (Ø—Ø), or different concentrations of 3-hydroxytamoxifen (●—●) in the absence (**a**) or presence (**b**) of $2 \times 10^{-10}$ M oestradiol for the indicated lengths of time. The data are expressed as described in the legend to Figure 2. **c**, MCF-7 cells were withdrawn and then cultured in the indicated concentrations of 3-hydroxytamoxifen in the presence (●—●) or absence (O—O) of $2 \times 10^{-10}$ M oestradiol. The levels of the pNR-1, pNR-2 pNR-25 and cathepsin D RNAs were measured as described in the legend to Figure 2c.

**Figure 7** Effect of metabolite E in MCF-7 cells. **a** and **b**, Withdrawn cells were cultured in the withdrawal medium alone (○—○), or containing $10^{-8}$ M (**a**) or $2 \times 10^{-10}$ M (**b**) oestradiol (∅—∅), or different concentrations of metabolite E (●—●) in the absence (**a**) or presence (**b**) of $2 \times 10^{-10}$ M oestradiol for the indicated lengths of time. The data are expressed as described in the legend to Figure 2. **c**, MCF-7 cells were withdrawn and then cultured in the indicated concentrations of metabolite E (○—○). The levels of the pNR-1, pNR-2, pNR-25 and cathepsin D RNAs were measured as described in the legend to Figure 2c.

**Figure 8** Effect of oestradiol and tamoxifen on the synthesis of cathepsin D. MCF-7 were plated into 8 mm diameter wells, withdrawn for 5 days and then cultured in withdrawal medium alone (---), $10^{-8}$ M oestradiol (---), the indicated concentration of tamoxifen alone (○—○) or $2\times10^{-10}$ M oestradiol together with the indicated concentration of tamoxifen (●—●). Cathepsin D synthesis was determined as described in **Materials and methods** and is expressed as a percentage of that in oestrogen-stimulated cells.

and all three compounds inhibited the oestrogen-induced proliferation in a dose-dependent manner.

The relative binding affinities of the four compounds for the oestrogen receptor in MCF-7 cells or rat uterus (Coezy *et al.*, 1982; Furr & Jordan, 1984; Roos *et al.*, 1983) agree well with the doses at which they inhibit oestrogen-induced growth. For instance at $10^{-6}$ M, 4-hydroxytamoxifen completely blocked the effect of oestrogen, 3-hydroxytamoxifen reduced it by 75%, tamoxifen by 25%, and *N*-desmethyltamoxifen was ineffective. In contrast, the degree to which they acted as oestrogens was not related to their affinity for the oestrogen receptor.

Berthois *et al.* (1986) reported that tamoxifen and 4-hydroxytamoxifen stimulate cell growth in phenol red-free medium at concentrations of $10^{-10}$ M and $10^{-8}$ M respectively. In a subsequent study (Katzenellenbogen *et al.*, 1987), a more extended range of concentrations was used and growth stimulation was observed at $10^{-10}$ and $10^{-9}$ M hydroxytamoxifen and at $10^{-10}$ to $10^{-7}$ M tamoxifen. Our data are therefore in broad agreement with these two studies except that we did not observe stimulation at $10^{-8}$ M tamoxifen.

The growth stimulation of the MCF-7 cells by metabolite E was near maximal at $10^{-9}$ M. This is a lower concentration than would have been predicted from its relative binding affinity for the rat uterine oestrogen receptor (Furr & Jordan, 1984) and suggests that the affinity of metabolite E for the human oestrogen receptor may be higher than reported. Different concentrations of metabolite E had no effect on the cell proliferation induced by a maximally stimulating concentration of oestradiol ($2\times10^{-10}$ M). The complete absence of any additive effect of these two compounds on cell growth indicates that they act by the same mechanism.

The stimulatory effects of tamoxifen and its metabolites on the growth of breast cancer cells has clear implications for the treatment of advanced carcinoma of the breast with tamoxifen. The major metabolites of tamoxifen detected in patients are *N*-desmethyltamoxifen and 4-hydroxytamoxifen (Robinson & Jordan, 1988). The mean tumour concentrations in women on tamoxifen (40 mg daily) are 25.1 ng mg protein$^{-1}$ for tamoxifen, 52 ng mg protein$^{-1}$ for *N*-desmethyltamoxifen, and 0.53 ng mg protein$^{-1}$ for 4-hydroxytamoxifen (Daniel *et al.*, 1981).

At these concentrations, which are in approximately inverse proportion to the relative binding affinities of the three anti-oestrogens for the oestrogen receptor, they could all affect the growth of oestrogen-responsive tumour cells. In the absence of endogenous oestrogens, the relative concentrations of these three anti-oestrogens would determine the growth response of the tumour cells. It is also possible that the tumour flare observed in some patients occurs during the transitory period in which tamoxifen or *N*-desmethyltamoxifen are present at higher concentrations before the accumulation of significant concentrations of 4-hydroxytamoxifen (Milano *et al.*, 1987).

In the presence of oestrogens, the growth of all oestrogen-responsive tumour cells would be inhibited by the three anti-oestrogens. Maximal growth inhibition would be dependent on the concentrations attained in the tumour cells.

Metabolite E was originally identified in bile but has been found as a minor metabolite in human plasma (Murphy *et al.*, 1987). It has not been detected in breast tumour cells. In the absence of any data on the intra-tumour concentrations of metabolite E, it is not possible to assess whether it has a significant oestrogenic effect in tumours of patients receiving tamoxifen. Because it is biologically active at a 100-fold lower concentration than tamoxifen, there may be biologically active concentrations in tumours that have escaped detection due to their low levels relative to tamoxifen and the major metabolites. Whatever the situation *in vivo*, these experiments establish the principle that *in vivo* metabolites of anti-oestrogens can be full oestrogens for the growth stimulation of human breast cancer cells.

The effects of the five anti-oestrogens on the levels of the four oestrogen-regulated RNAs also varied. As with cell growth, metabolite E was a full oestrogen for the induction of all four RNAs. The oestrogenicity of the other anti-oestrogens for the induction of the RNAs appeared specific for each RNA. All four induced the pNR-1 RNA above the level induced by oestrogen. Cathepsin D RNA was induced to the same level by oestrogen and three of the four anti-oestrogens. The pNR-2 and pNR-25 RNA levels were less affected by the anti-oestrogens. Although the maximal effect of the drugs was not related to their affinity for the oestrogen receptor, the concentration at which they were active was (see Table I for summary).

These results are in broad agreement with, but substantially extend, a previous study in which the effects of tamoxifen and 4-hydroxytamoxifen on pNR-1 and pNR-2 RNA levels were measured (May & Westley, 1987). In that study, tamoxifen and 4-hydroxytamoxifen induced the pNR-1 RNA levels to 80% of the level in oestradiol-treated cells. The induction by tamoxifen in the present study was even greater (2-fold) than that of oestradiol. Two different sublines of MCF-7 cells were used in the two studies. The subline used in this study was chosen because it showed a better proliferative response to oestradiol. These results suggest that it is also more responsive to tamoxifen and its metabolites.

Tamoxifen, *N*-desmethyltamoxifen, 4-hydroxytamoxifen and 3-hydroxytamoxifen all inhibited the induction of the pNR-2 and pNR-25 RNAs by oestradiol. The relative concentrations required for half-maximal inhibition were inversely proportional to their relative affinities for the oestrogen receptor. Interestingly in the presence of oestrogen, although tamoxifen and its derivatives induced the pNR-1 RNA levels to the same level as in the absence of oestrogen, a higher concentration of the drug was required. It appears

**Table I** Summary of effects of anti-oestrogens on cell growth and the four oestrogen-regulated RNAs

| | *Growth* | *pNR-1* | *pNR-2* | *pNR-25* | *Cathepsin D* |
|---|---|---|---|---|---|
| *Tamoxifen* | | | | | |
| Induction: | | | | | |
| % of oestradiol | 20 | 200 | 30 | 15 | 100 |
| Concentration (log molar) | −7 | −7 | −6 | −7 | −7 |
| Inhibition: | | | | | |
| concentration (log molar) | −5 | No inhibition | −5 | −5 | No inhibition |
| N-*desmethyltamoxifen* | | | | | |
| Induction: | | | | | |
| %of oestradiol | 25 | 200 | No induction | 18 | 30 |
| Concentration (log molar) | −7 | −7 | | −7 | −7 |
| Inhibition: | | | | | |
| concentration (log molar) | −5 | No inhibition | −5 | −5 | No inhibition |
| *4-hydroxytamoxifen* | | | | | |
| Induction: | | | | | |
| % of oestradiol | 8 | 200 | No induction | No induction | 100 |
| Concentration (log molar) | −9 | −9 | | | −9 |
| Inhibition: | | | | | |
| concentration: (log molar) | −6 | No inhibition | −6 | −6 | No inhibition |
| *3-hydroxytamoxifen* | | | | | |
| Induction: | | | | | |
| % of oestradiol | 13 | 150 | 22 | 10 | 100 |
| Concentration (log molar) | −8 | −8 | −9 | −8 | −7 |
| Inhibition: | | | | | |
| concentration (log molar) | −5 | No inhibition | −5 | −5 | No inhibition |
| *Metabolite E* | | | | | |
| % of oestradiol | 100 | 100 | 100 | 100 | 100 |
| Concentration (log molar) | −7 | −8 | −7 | −7 | −8 |

For each anti-oestrogen, the maximum induction (expressed as a percentage of the oestrogen induced level) is shown together with the concentration required for maximal induction. The concentration required to inhibit the induction by 0.2 nM oestradiol is also given.

that oestrogen is acting as a tamoxifen antagonist for the induction of the pNR-1 RNA.

The effect of tamoxifen and its derivatives on the levels of cathepsin D RNA in the presence of oestrogen was more bizarre (Figure 3). The induction followed a bell-shaped dose–response curve. The stimulation was greatest for tamoxifen and least marked for *N*-desmethyltamoxifen. The relative concentrations of the four drugs that gave maximum stimulation agreed with their relative affinities for the oestrogen receptor.

As these effects were so dramatic, cathepsin D synthesis was measured in cells cultured under the same conditions as in the RNA experiments. Similar dose–response curves were obtained and cathepsin D synthesis was more than 3-fold higher in cells treated with oestradiol and 1–5 x $10^{-6}$ M tamoxifen than in cells treated with oestradiol alone. This shows that the alterations in cathepsin D mRNA levels effected by small changes in the relative concentrations of tamoxifen and oestradiol can cause a large difference in the level of cathepsin D synthesis.

The mechanisms involved in this synergistic effect are currently unclear. At low concentrations of the anti-oestrogen, most of the receptor would be complexed with oestradiol whereas at higher concentrations of the anti-oestrogens, some oestrogen receptors would be complexed with oestradiol and some with the anti-oestrogen. In this situation, there is a greater induction of the cathepsin D gene than in the presence of oestradiol alone. At high concentrations of the anti-oestrogens, all of the oestrogen receptor molecules will be complexed with anti-oestrogen and the expression of the cathepsin D gene is as found in cells treated with the anti-oestrogen alone.

Steroids are thought to regulate gene expression by an interaction of the receptor complex with regulatory DNA sequences and it has been suggested that the oestrogen receptor binds to oestrogen response elements as a dimer (Kumar & Chambon, 1988). It is therefore possible that an oestrogen receptor dimer complexed with one oestradiol and one anti-oestrogen molecule is interpreted by the cathepsin D response element as being more oestrogenic than a receptor dimer complexed with two oestradiol or two anti-oestrogen molecules.

The combined effect of tamoxifen and oestradiol on the induction of cathepsin D RNA could have clinical implications. Although the biological role of cathepsin D in breast tumours is not known, it has been proposed to be involved in tumour dissemination as it can be secreted from breast tumour cells (Westley & Rochefort, 1980) and can degrade extracellular matrix (Briozzo *et al.*, 1988). The present observations suggest that tamoxifen treatment might enhance any tumour dissemination mediated by cathepsin D. However, cathepsin D is also thought to be involved in tissue involution and remodelling; and in processes such as post-partum involution of the uterus, cathepsin D levels are under some degree of hormonal control (Afting et al., 1979). Cathepsin D may therefore play an active role in tumour regression and the dramatic elevation of cathepsin D by tamoxifen in the presence of oestradiol might facilitate this process. This model would predict that high levels of cathepsin D in primary tumours should be associated with a good prognosis. In this context, a recent immunohistochemical study has shown that high levels of cathepsin D in breast cancer cells is indicative of a good prognosis in oestrogen receptor positive tumours (J.A. Henry, personal communication). It will be interesting to determine how patients who express high tumour levels of cathepsin D respond to tamoxifen therapy.

A previous study did not detect a synergistic effect between oestradiol and tamoxifen on the secretion of cathepsin D from MCF-7 cells (Westley & Rochefort, 1980). The discrepancy between the two studies may be accounted for by differences in experimental protocols, such as the use of phenol red-free medium or the more detailed dose–response curves obtained in the present study. Alternatively, the two MCF-7 sublines used may respond differently. This is currently being investigated.

Tamoxifen and its metabolites have extremely variable effects on cell proliferation and the levels of oestrogen-inducible RNAs in MCF-7 cells. Comparison of the effects of each compound on cell growth and RNA expression should identify those RNAs whose levels are correlated with cell growth. Such an RNA might be valuable as a marker for predicting and/or monitoring responses to endocrine therapy and might itself be implicated in the growth response.

The regulation of the pNR-2 and pNR-25 RNAs are most closely allied to cell growth (Table I). The growth stimulation and regulation of the two RNAs by tamoxifen, 3-hydroxytamoxifen and metabolite E is similar and occurs at approximately the same concentration for each anti-oestrogen. The concentrations at which all four anti-oestrogens inhibit the induction by oestradiol of cell growth and the levels of the pNR-2 and pNR-25 RNAs also agree well. However, although *N*-desmethyltamoxifen does not affect the levels of the pNR-2 RNA, it does stimulate MCF-7 cell proliferation and increase the levels of the pNR-25 RNA. We are currently evaluating these two RNAs as predictive markers of response to anti-oestrogen therapy.

This work was supported by the North of England Cancer Research Campaign, the Gunnar Nilsson Cancer Research Trust Fund, the Medical Research Council and ICI plc. F.E.B. May thanks the Royal Society for a 1983 University Research Fellowship. We thank ICI pharmaceuticals plc for the gift of tamoxifen, *N*-desmethyltamoxifen, 4-hydroxytamoxifen and metabolite E, and Klinge Pharma GmbH for the gift of 3-hydroxytamoxifen.

### References

AFTING, E.G., BECKER, M.L. & ELCE, J.S. (1979). Proteinase and proteinase-inhibitor activities of rat uterine myometrium during pregnancy and involution. *Biochem. J.*, **177**, 99.

BERTHOIS, Y., KATZENELLENBOGEN, J.A. & KATZENELLENBOGEN, B.S. (1986). Phenol red in tissue culture media is a weak estrogen: implications concerning the study of estrogen-responsive cells in culture. *Proc. Natl Acad. Sci. USA*, **83**, 2496.

BRIOZZO, P., MORISSET, M., CAPONY, F., ROUGEOT, C. & ROCHEFORT, H. (1988). *In vitro* degradation of extracellular matrix with Mr52,000 cathepsin D secreted by breast cancer cells. *Cancer Res.*, **48**, 3688.

BUETTI, E. & DIGGELMANN, H. (1981). Cloned mouse mammary tumour virus DNA is biologically active in transfected mouse cells and its expression is stimulated by glucocorticoid hormones. *Cell*, **23**, 335.

COEZY, E., BORGNA, J.-L. & ROCHEFORT, H. (1982). Tamoxifen and metabolites in MCF-7 cells: correlation between binding to estrogen receptor and inhibition of cell growth. *Cancer Res.*, **42**, 317.

DANIEL, P., GASKELL, S.J., BISHOP, C.C. & NICHOLSON, R.I. (1981). Determination of tamoxifen and an hydroxylated metabolite in plasma from patients with advanced breast cancer using gas chromatography-mass spectrometry. *Eur. J. Cancer Clin. Oncol.*, **17**, 1183.

DOWNS, T.R. & WILFINGER, W.W. (1983). Fluorometric quantification of DNA in cells and tissue. *Anal. Biochem.*, **131**, 538.

FURR, B.J.A. & JORDAN, V.C. (1984). The pharmacology and clinical uses of tamoxifen. *Pharmaceut. Ther.*, **25**, 127.

HORWITZ, K., ZAVA, D.T., THILAGAR, A.K., JENSEN, E.M. & McGUIRE, W.L. (1978). Steroid receptor analyses of nine human breast cancer cell lines. *Cancer Res.*, **38**, 2434.

KATZENELLENBOGEN, B.S., KENDRA, K.L., NORMAN, M.J. & BERTHOIS, Y. (1987). Proliferation, hormonal responsiveness, and estrogen receptor content of MCF-7 human breast cancer cells grown in the short-term and long-term absence of estrogens. *Cancer Res.*, **47**, 4355.

KUMAR, V. & CHAMBON, P. (1988). The estrogen receptor binds tightly to its responsive element as a ligand-induced homodimer. *Cell*, **55**, 145.

LASKEY, R.A. & MILLS, A.D. (1975). Quantitative film detection of $^3H$ and $^{14}C$ in polyacrylamide gels by fluorography. *Eur. J. Biochem.*, **56**, 335.

LIPPMAN, M.E., BOLAN, G. & HUFF, K.K. (1976). The effects of estrogens and antiestrogens on hormone responsive breast cancer in lo[illegible] term tissue culture. *Cancer Res.*, **36**, 4595.

MASIAK[illegible]SKI, P., BREATHNACH, R., BLOCH, J., GANNON, F., KRUST, A. & CHAMBON, P. (1982). Cloning of cDNA sequences of hormone-regulated genes from the MCF-7 human breast cancer cell line. *Nucleic Acids Res.*, **10**, 7895.

MAY, F.E.B. & WESTLEY, B.R. (1986). Cloning of estrogen regulated messenger RNA from human breast cancer cells. *Cancer Res.*, **46**, 6034.

MAY, F.E.B. & WESTLEY, B.R. (1987). Effects of tamoxifen and 4-hydroxytamoxifen on the pNR-1 and pNR-2 estrogen-regulated RNAs in human breast cancer cells. *J. Biol. Chem.*, **262**, 15894.

MAY, F.E.B. & WESTLEY, B.R. (1988). Identification and characterisation of estrogen-regulated RNAs in human breast cancer cells. *J. Biol. Chem.*, **263**, 12901.

MELTON, M.A., KRIEG, P.A., REBAGLIATI, M.R., MANIATIS, T., ZINN, K. & GREEN, G.R. (1984). Efficient *in vitro* synthesis of biologically active RNA and RNA hybridisation probes from plasmids containing a bacteriophage SP6 promoter. *Nucleic Acids Res.*, **12**, 7035.

MILANO, G., ETIENNE, M.C., FRENAY, M. and 7 others (1987) Optimised analysis of tamoxifen and its main metabolites in the plasma and cytosol of mammary tumours. *Br. J. Cancer*, **55**, 509.

MURPHY, C., FOTSIS, T., PANTZAR, P., ALDERCREUTZ, H. & MARTIN, F. (1987). Analysis of tamoxifen and its metabolites in human plasma by gas chromatography-mass spectrometry (GC-MS) using selected ion monitoring (SIM). *J. Steroid Biochem.*, **26**, 547.

REID, W.A., VALLER, M.J. & KAY, J. (1986). Immunolocalisation of cathepsin D in normal and neoplastic human tissues. *J. Clin. Pathol.*, **39**, 1323.

RIO, M.C., BELLOCQ, J.P., GAIRARD, B. and 7 others (1987). Specific expression of the pS2 gene in subclasses of breast cancers in comparison with expression of the estrogen and progesterone receptors and the oncogene ERBB2. *Proc. Natl Acad. Sci. USA*, **84**, 9243.

RIO, M.C., BELLOCQ, J.P., DANIEL, J.Y. and 5 others (1988). Breast cancer-associated pS2 protein: synthesis and secretion by normal stomach mucosa. *Science*, **241**, 705.

ROBINSON, S.O. & JORDAN, V.C. (1988). Metabolism of steroid-modifying anticancer agents. *Pharmaceut. Ther.*, **36**, 41.

ROOS, W., OEZE, L., LOSER, R. & EPPENBERGER, U. (1983). Antiestrogenic action of 3-hydroxytamoxifen in the human breast cancer cell line MCF-7. *J. Natl Cancer Inst.*, **71**, 55.

SOULE, H.D., VASQUEZ, J., LANG, A., ALBERT, S. & BRENNAN, M.A. (1973). A human cell line from a pleural effusion derived from a breast carcinoma. *J. Natl Cancer Inst.*, **51**, 1409.

WESTLEY, B.R. & MAY, F.E.B. (1987). Oestrogen regulates cathepsin D mRNA levels in oestrogen responsive human breast cancer cells. *Nucleic Acids Res.*, **15**, 3773.

WESTLEY, B. & ROCHEFORT, H. (1980). A secreted glycoprotein induced by estrogens in human breast cancer cell lines. *Cell*, **20**, 353.


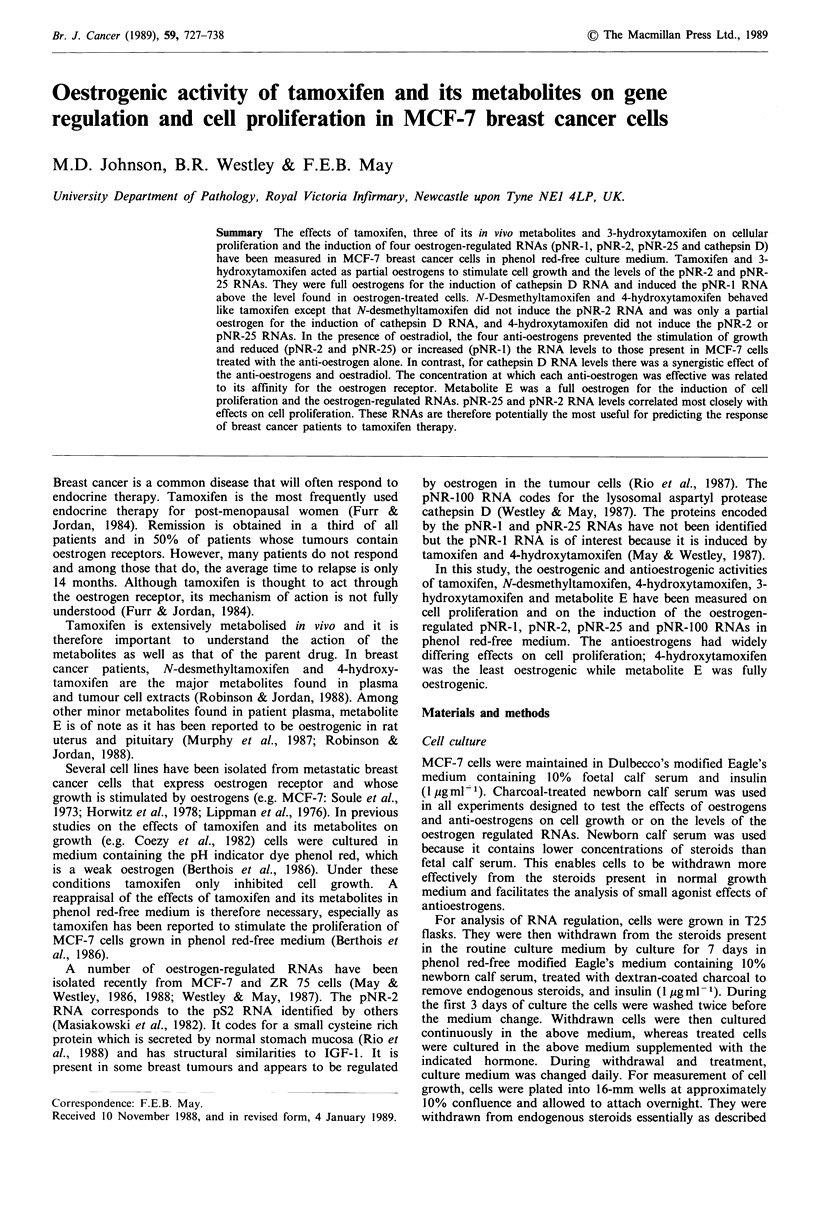

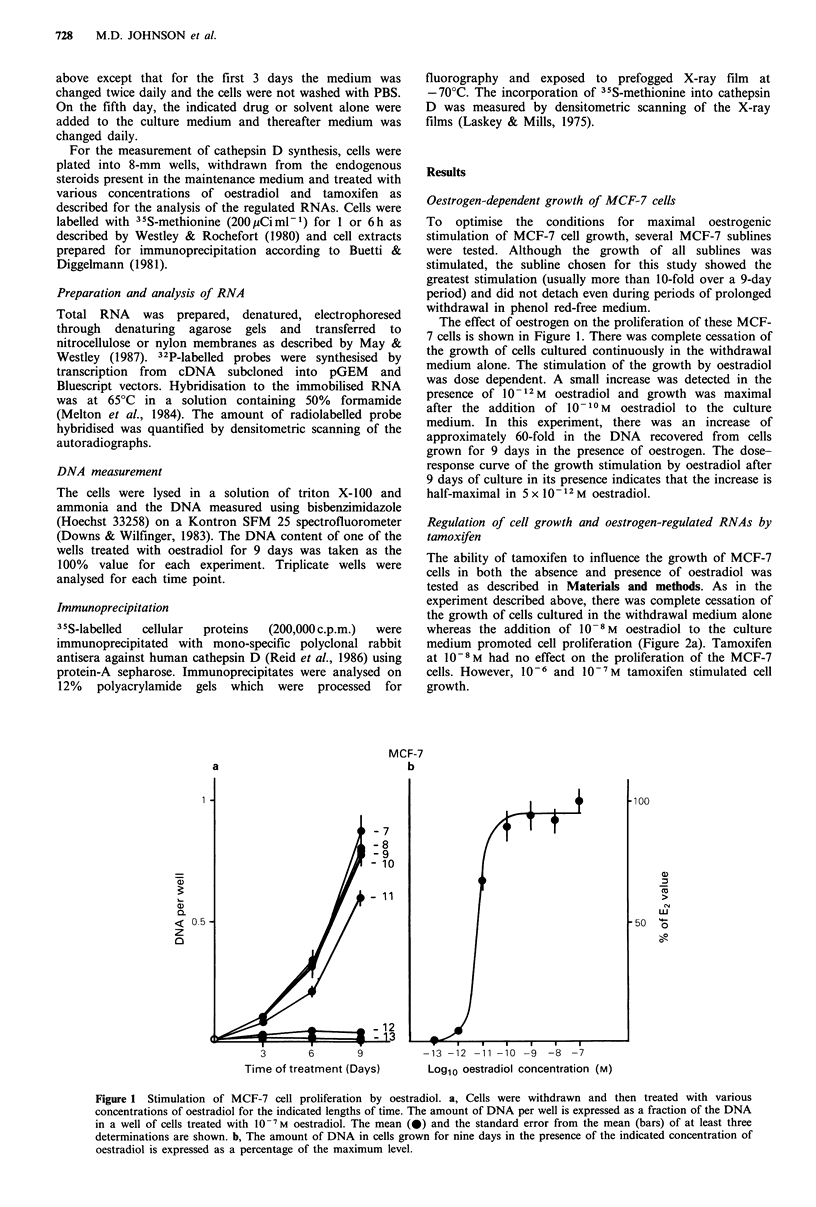

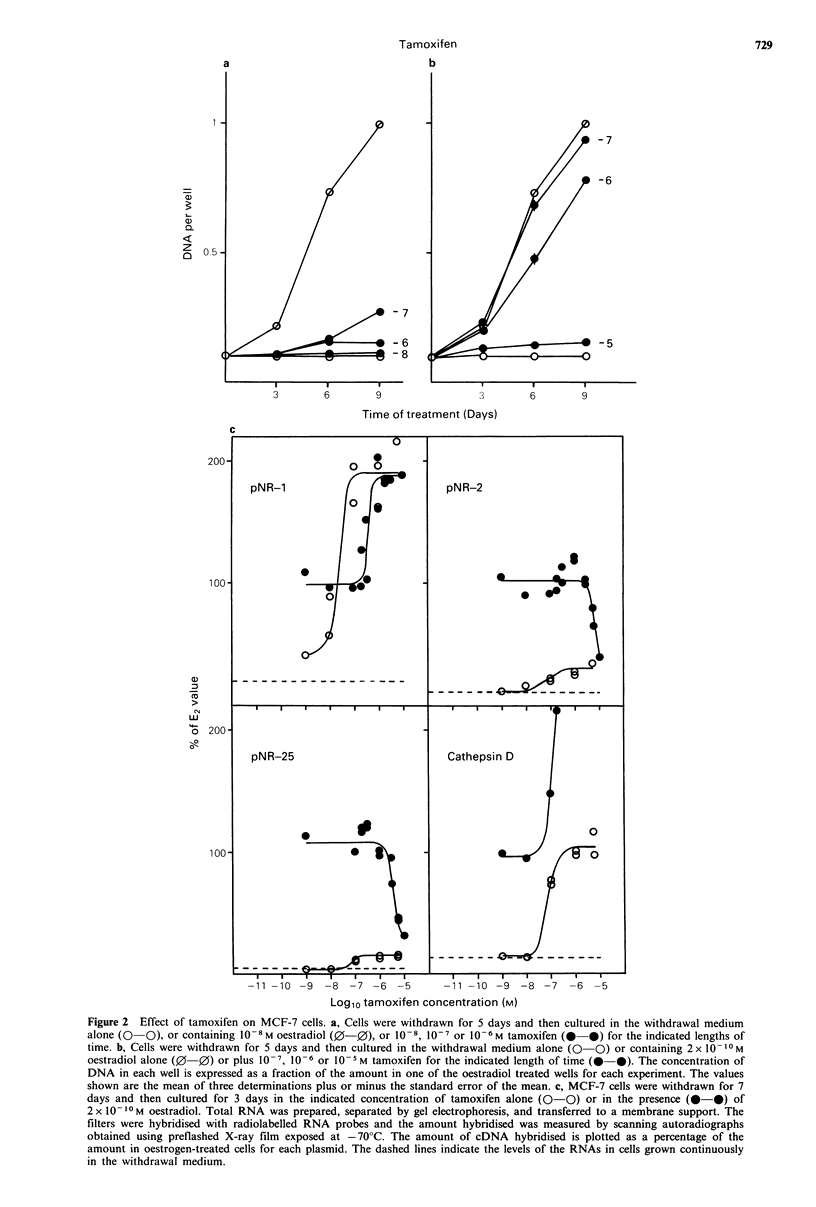

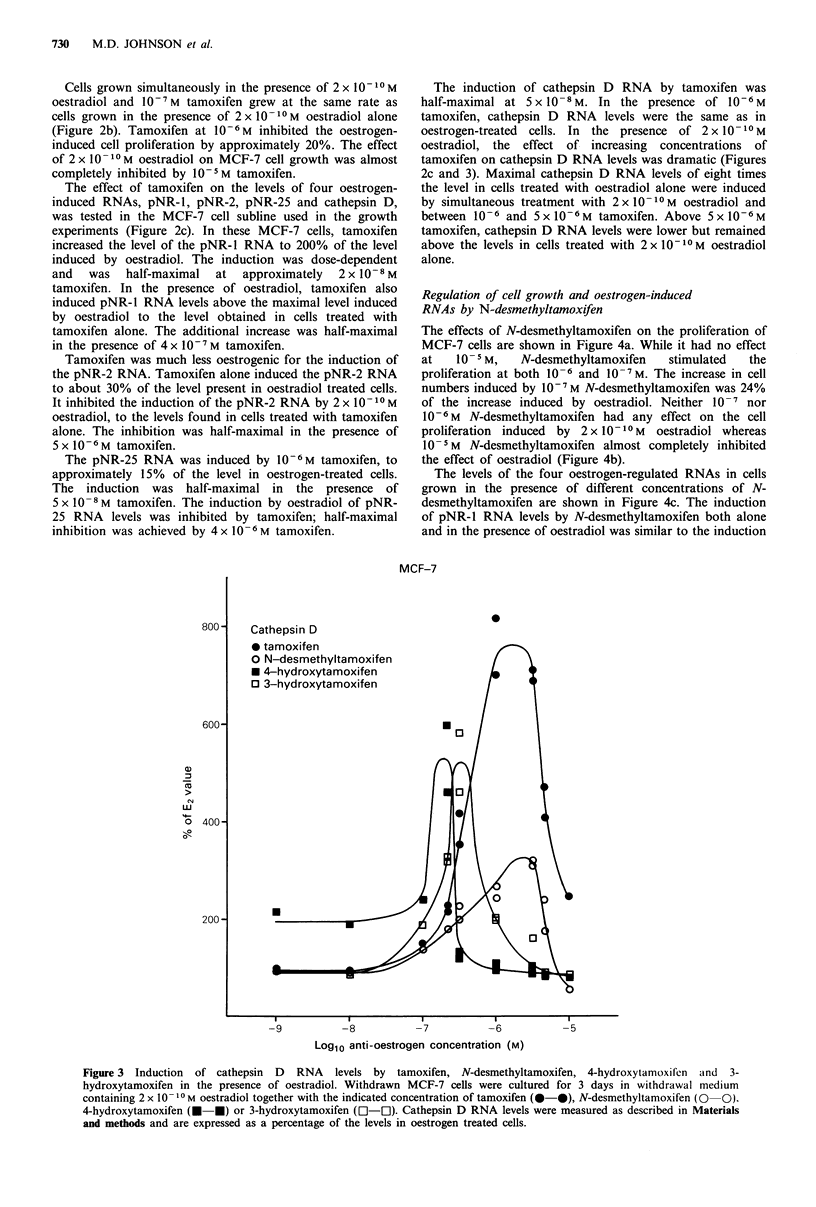

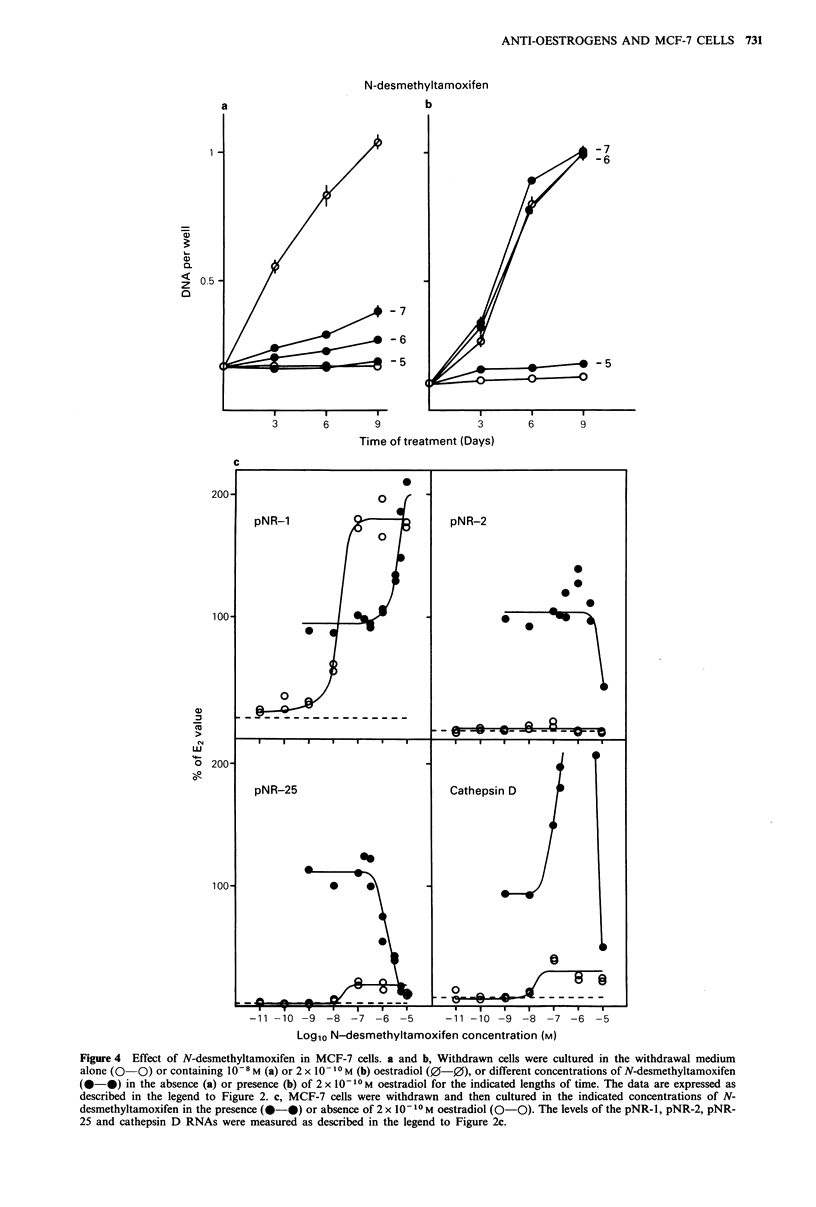

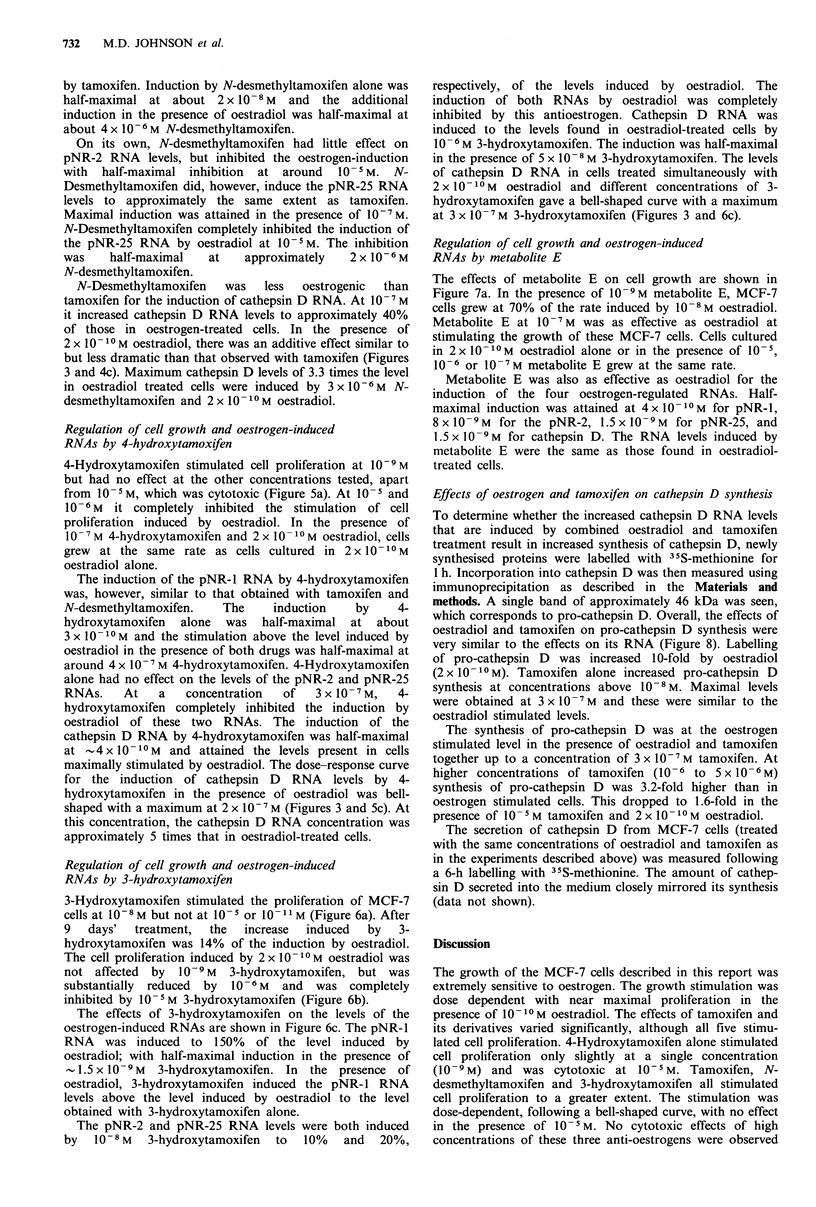

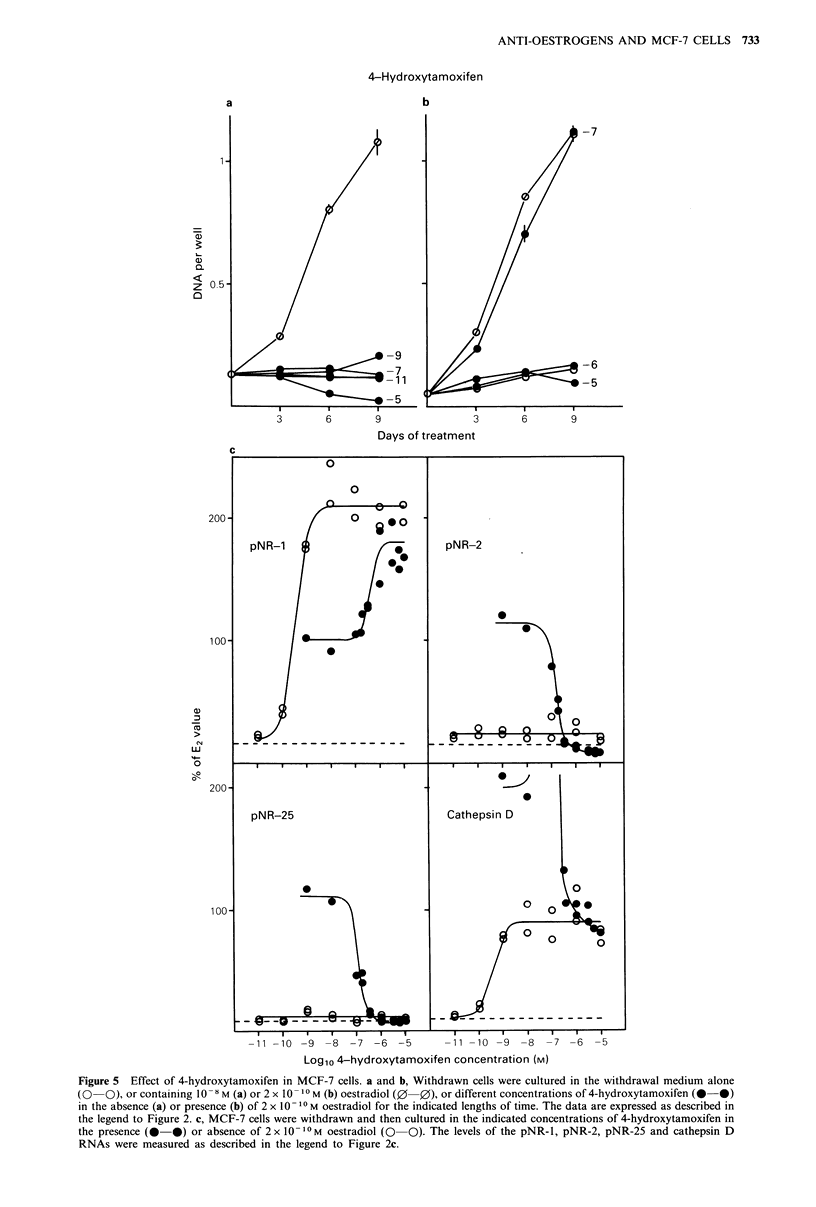

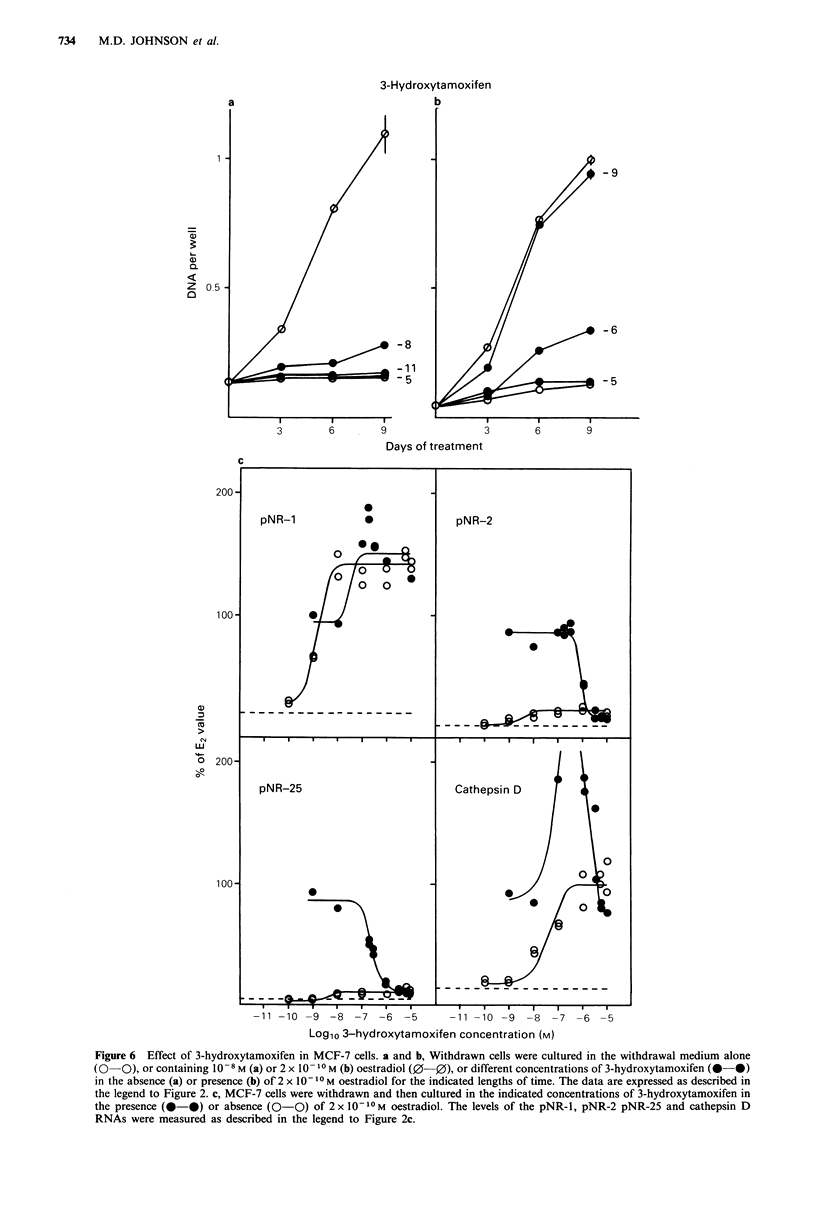

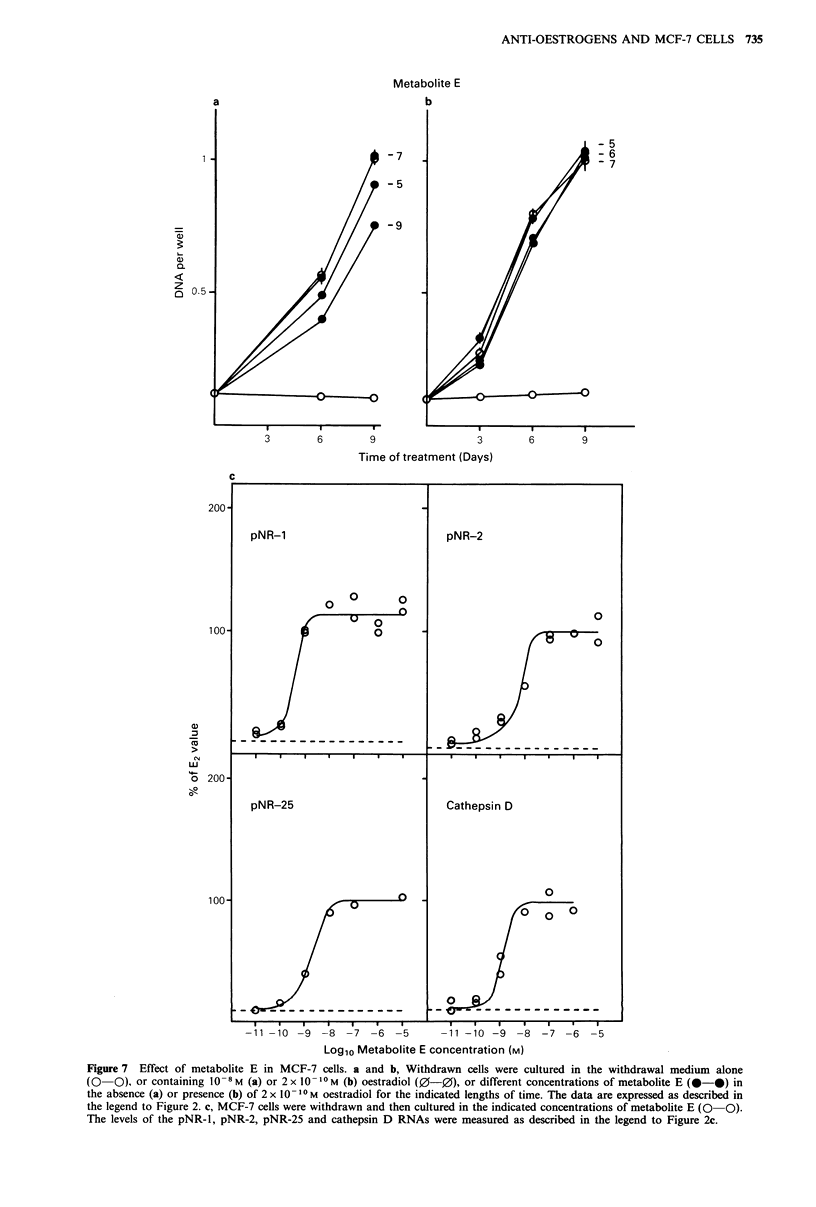

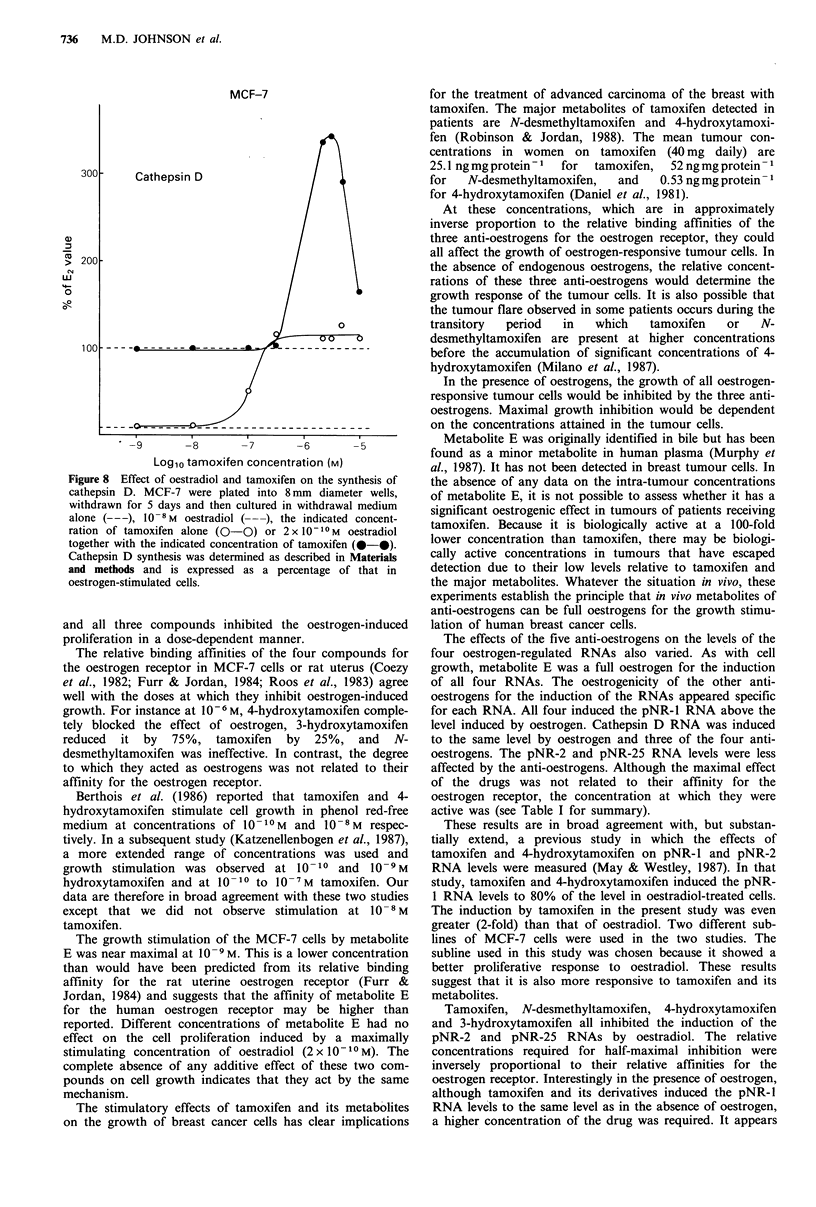

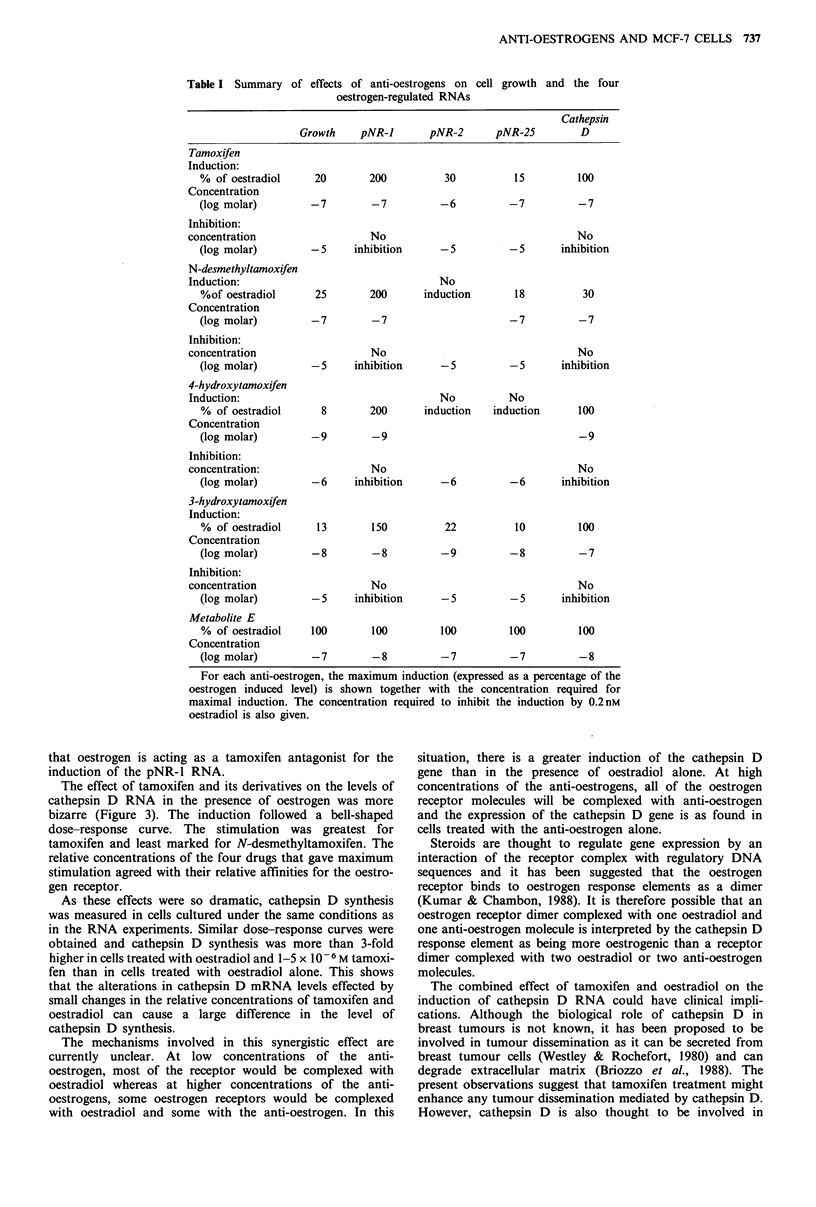

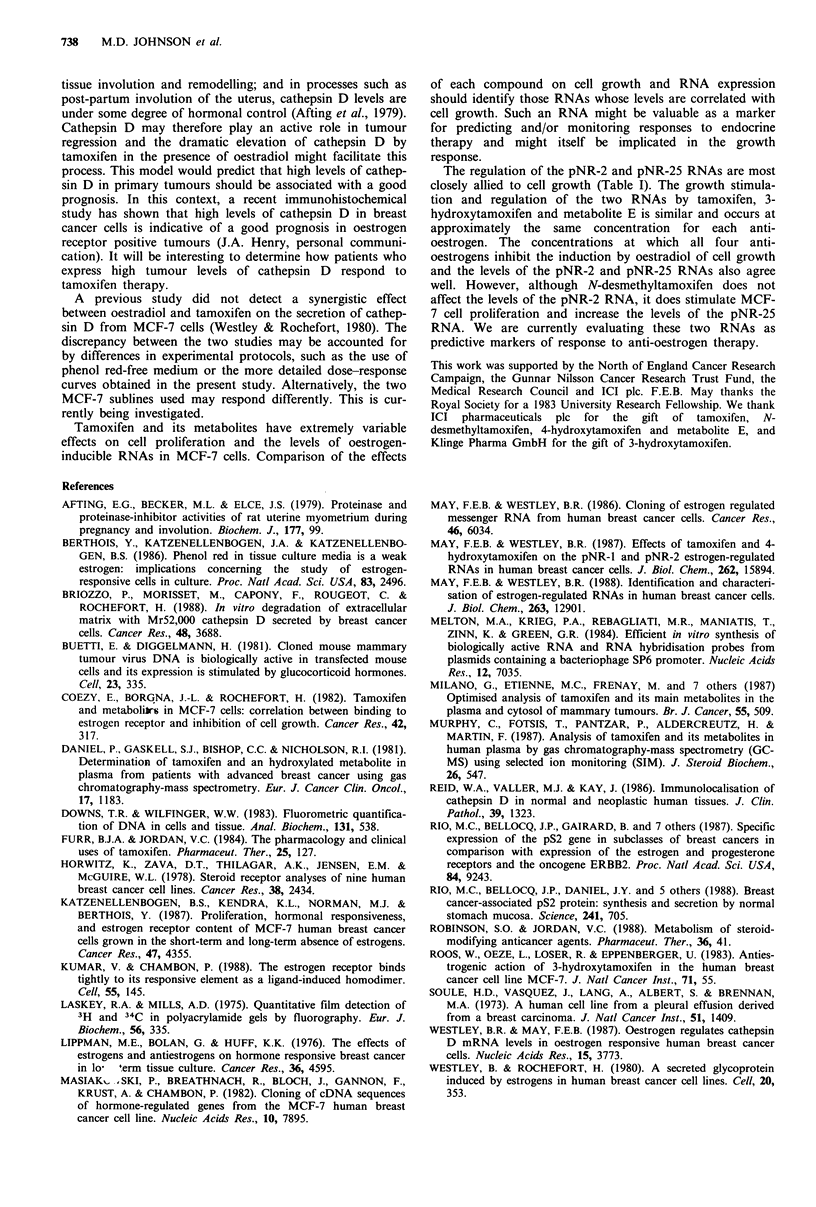


## References

[OCR_01194] Afting E. G., Becker M. L., Elce J. S. (1979). Proteinase and proteinase-inhibitor activities of rat uterine myometrium during pregnancy and involution.. Biochem J.

[OCR_01201] Berthois Y., Katzenellenbogen J. A., Katzenellenbogen B. S. (1986). Phenol red in tissue culture media is a weak estrogen: implications concerning the study of estrogen-responsive cells in culture.. Proc Natl Acad Sci U S A.

[OCR_01204] Briozzo P., Morisset M., Capony F., Rougeot C., Rochefort H. (1988). In vitro degradation of extracellular matrix with Mr 52,000 cathepsin D secreted by breast cancer cells.. Cancer Res.

[OCR_01210] Buetti E., Diggelmann H. (1981). Cloned mouse mammary tumor virus DNA is biologically active in transfected mouse cells and its expression is stimulated by glucocorticoid hormones.. Cell.

[OCR_01216] Coezy E., Borgna J. L., Rochefort H. (1982). Tamoxifen and metabolites in MCF7 cells: correlation between binding to estrogen receptor and inhibition of cell growth.. Cancer Res.

[OCR_01222] Daniel P., Gaskell S. J., Bishop H., Campbell C., Nicholson R. I. (1981). Determination of tamoxifen and biologically active metabolites in human breast tumours and plasma.. Eur J Cancer Clin Oncol.

[OCR_01229] Downs T. R., Wilfinger W. W. (1983). Fluorometric quantification of DNA in cells and tissue.. Anal Biochem.

[OCR_01233] Furr B. J., Jordan V. C. (1984). The pharmacology and clinical uses of tamoxifen.. Pharmacol Ther.

[OCR_01237] Horwitz K. B., Zava D. T., Thilagar A. K., Jensen E. M., McGuire W. L. (1978). Steroid receptor analyses of nine human breast cancer cell lines.. Cancer Res.

[OCR_01242] Katzenellenbogen B. S., Kendra K. L., Norman M. J., Berthois Y. (1987). Proliferation, hormonal responsiveness, and estrogen receptor content of MCF-7 human breast cancer cells grown in the short-term and long-term absence of estrogens.. Cancer Res.

[OCR_01249] Kumar V., Chambon P. (1988). The estrogen receptor binds tightly to its responsive element as a ligand-induced homodimer.. Cell.

[OCR_01254] Laskey R. A., Mills A. D. (1975). Quantitative film detection of 3H and 14C in polyacrylamide gels by fluorography.. Eur J Biochem.

[OCR_01259] Lippman M., Bolan G., Huff K. (1976). The effects of estrogens and antiestrogens on hormone-responsive human breast cancer in long-term tissue culture.. Cancer Res.

[OCR_01266] Masiakowski P., Breathnach R., Bloch J., Gannon F., Krust A., Chambon P. (1982). Cloning of cDNA sequences of hormone-regulated genes from the MCF-7 human breast cancer cell line.. Nucleic Acids Res.

[OCR_01270] May F. E., Westley B. R. (1986). Cloning of estrogen-regulated messenger RNA sequences from human breast cancer cells.. Cancer Res.

[OCR_01275] May F. E., Westley B. R. (1987). Effects of tamoxifen and 4-hydroxytamoxifen on the pNR-1 and pNR-2 estrogen-regulated RNAs in human breast cancer cells.. J Biol Chem.

[OCR_01279] May F. E., Westley B. R. (1988). Identification and characterization of estrogen-regulated RNAs in human breast cancer cells.. J Biol Chem.

[OCR_01284] Melton D. A., Krieg P. A., Rebagliati M. R., Maniatis T., Zinn K., Green M. R. (1984). Efficient in vitro synthesis of biologically active RNA and RNA hybridization probes from plasmids containing a bacteriophage SP6 promoter.. Nucleic Acids Res.

[OCR_01291] Milano G., Etienne M. C., Frenay M., Khater R., Formento J. L., Renee N., Moll J. L., Francoual M., Berto M., Namer M. (1987). Optimised analysis of tamoxifen and its main metabolites in the plasma and cytosol of mammary tumours.. Br J Cancer.

[OCR_01295] Murphy C., Fotsis T., Pantzar P., Adlercreutz H., Martin F. (1987). Analysis of tamoxifen and its metabolites in human plasma by gas chromatography-mass spectrometry (GC-MS) using selected ion monitoring (SIM).. J Steroid Biochem.

[OCR_01302] Reid W. A., Valler M. J., Kay J. (1986). Immunolocalization of cathepsin D in normal and neoplastic human tissues.. J Clin Pathol.

[OCR_01314] Rio M. C., Bellocq J. P., Daniel J. Y., Tomasetto C., Lathe R., Chenard M. P., Batzenschlager A., Chambon P. (1988). Breast cancer-associated pS2 protein: synthesis and secretion by normal stomach mucosa.. Science.

[OCR_01307] Rio M. C., Bellocq J. P., Gairard B., Rasmussen U. B., Krust A., Koehl C., Calderoli H., Schiff V., Renaud R., Chambon P. (1987). Specific expression of the pS2 gene in subclasses of breast cancers in comparison with expression of the estrogen and progesterone receptors and the oncogene ERBB2.. Proc Natl Acad Sci U S A.

[OCR_01319] Robinson S. P., Jordan V. C. (1988). Metabolism of steroid-modifying anticancer agents.. Pharmacol Ther.

[OCR_01323] Roos W., Oeze L., Löser R., Eppenberger U. (1983). Antiestrogenic action of 3-hydroxytamoxifen in the human breast cancer cell line MCF-7.. J Natl Cancer Inst.

[OCR_01328] Soule H. D., Vazguez J., Long A., Albert S., Brennan M. (1973). A human cell line from a pleural effusion derived from a breast carcinoma.. J Natl Cancer Inst.

[OCR_01333] Westley B. R., May F. E. (1987). Oestrogen regulates cathepsin D mRNA levels in oestrogen responsive human breast cancer cells.. Nucleic Acids Res.

[OCR_01338] Westley B., Rochefort H. (1980). A secreted glycoprotein induced by estrogen in human breast cancer cell lines.. Cell.

